# Acute Myocardial Infarction due to Coronary Artery Embolism in a Patient with Mechanical Aortic Valve Prosthesis

**DOI:** 10.1155/2010/751857

**Published:** 2010-06-14

**Authors:** Marcelo A. Nakazone, Bruno G. Tavares, Maurício N. Machado, Lilia N. Maia

**Affiliations:** ^1^Department of Cardiology and Cardiovascular Surgery, São José do Rio Preto Medical School, CEP 15090-000, São José do Rio Preto, SP, Brazil; ^2^Department of Molecular Biology, São José do Rio Preto Medical School, Av. Brigadeiro Faria Lima 5416, CEP 15090-000, São José do Rio Preto, SP, Brazil

## Abstract

Previous cases of coronary embolism as a cause of myocardial infarction (MI) in association with prosthetic mechanical valves have been reported, but the fact that the patient was not aware of the importance of maintaining anticoagulation therapy is relevant in this case. A 16-year-old female was referred for primary coronary intervention due to subacute anterolateral ST elevation MI, after she decided to discontinue warfarin therapy three weeks before. Coronary angiography showed distal occlusion of the left anterior descending coronary artery with an image suggesting embolic material. Conventional echocardiography demonstrated akinesia of anteroseptal, inferior, and posterior segments of the left ventricle, with severe systolic dysfunction, beyond the intraventricular thrombus. The presence of mechanic aortic prosthesis and no anticoagulation therapy are highly suggestive of coronary embolism as the cause of MI. This case report confirms that patient education is vital in our struggle to prevent this complication in high-risk patients.

## 1. Introduction

Prosthetic mechanical valves carry a significant thromboembolic risk justifying the use of long-term conventional anticoagulation therapy with warfarin [1–3]. The present report describes a case of embolic subacute anterolateral ST elevation myocardial infarction in a younger patient with aortic mechanical valve prosthesis who decided to discontinue warfarin without medical advice.

## 2. Case Report

 In October 2009, a 16-year-old female presented to the emergency department with sudden onset of severe chest pain, vomiting, and diaphoresis one day earlier. She had a history of rheumatic aortic stenosis and had received a 23 mm St. Jude mechanical aortic valve and mitral valve repair four years earlier. Although she had received medical advice regarding constant and imminent thromboembolic risk, she nonetheless decided to abruptly discontinue warfarin and started taking oral contraceptive pills three weeks earlier. She was a smoker but had no other risk factors for coronary artery disease.

**Figure 1:**
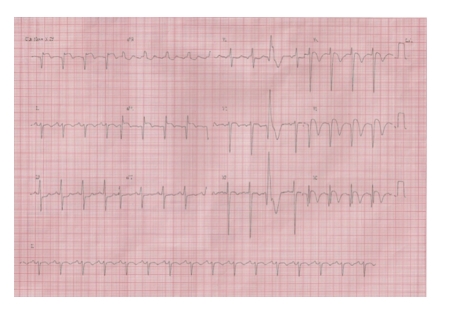
Electrocardiography showing left-axis deviation with ST elevation, T-wave inversion and Q-waves in leads I, aVL, and V5 to V6, compatible with subacute anterolateral myocardial infarction and a single ectopic beat.

**Figure 2:**
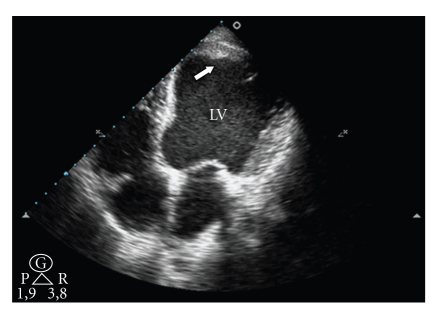
Transthoracic echocardiogram image showing thrombus sessile (white arrow) on left ventricular apex (LV).

At admission, the patient was tachypneic at 25 beats/min with regular rhythm at 107 beats/min. Her blood pressure was 154/92 mmHg. Cardiac auscultation was unchanged with normal prosthetic valve clicks and systolic ejection murmur grade 3/6. No crackles were heard over lungs. Electrocardiography showed left-axis deviation with ST elevation in leads I, aVL, and V5 to V6, compatible with anterolateral wall subacute myocardial infarction (Figure 1). Laboratory tests revealed cardiac troponin I >50 ng/mL (upper limit 0.01 ng/mL) and RNI of 1.4. Transthoracic echocardiography examination showed normally functioning aortic valve prosthesis with evidence of intraventricular thrombus (Figure 2) and akinesia of the anteroseptal, inferior, and posterior segments of the left ventricle.

**Figure 3:**
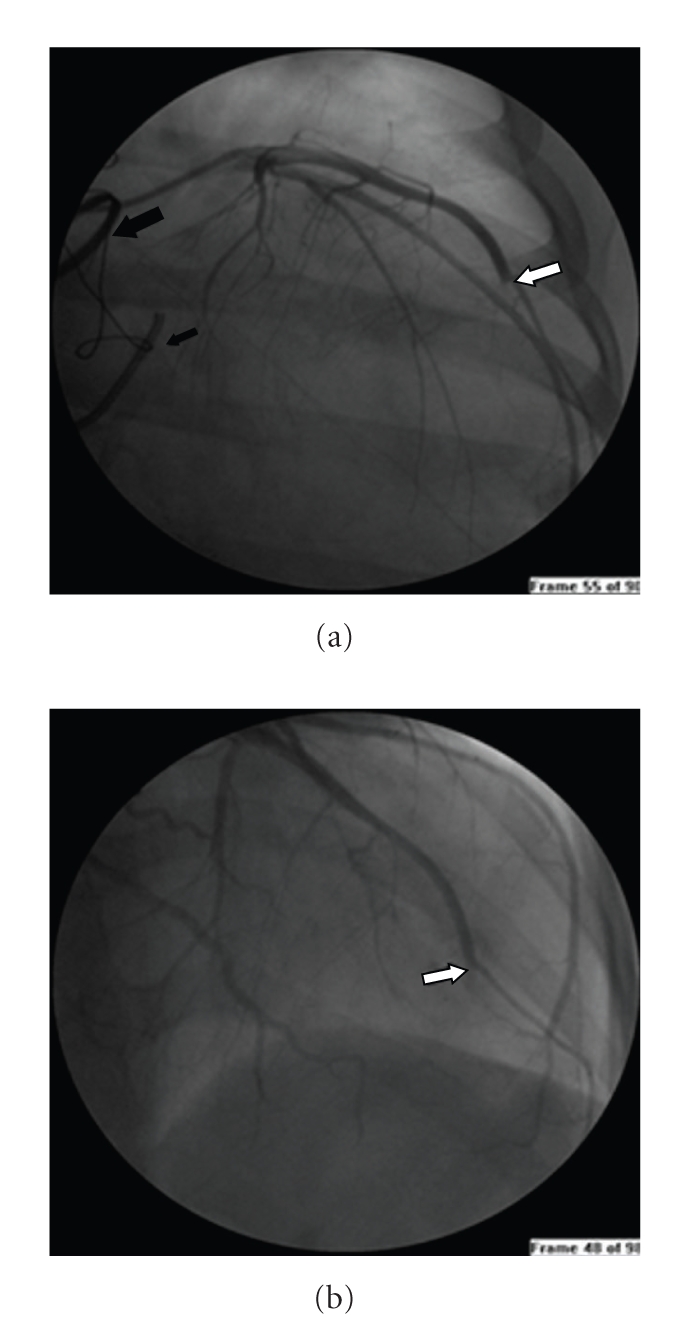
Coronary angiograms showing distal occlusion of the left anterior descending coronary artery with a concave endoluminal image strongly suggesting embolic material in two different projections (white arrows), mechanical aortic valve prosthesis (large black arrow) and mitral valve repair (small black arrow).

Oxygen, acetylsalicylic acid, clopidogrel, and heparin were administered, and the patient was transferred to the catheterization laboratory. Coronary angiography showed a distal occlusion of the left anterior descending coronary artery with a concave endoluminal image strongly suggesting embolic material (Figures 3(a) and 3(b)). The other coronary arteries were normal. Intracoronary catheter aspiration was attempted but was not successful. In addition, percutaneous coronary intervention was attempted but was also unsuccessful due to the distal location of coronary thrombus. We decided not to use thrombolytic therapy because there are fewer published reports and their results are not definitive. On Day 8, a transthoracic echocardiography only showed dyskinesia of the anterior and apical segments of the left ventricle and persistent image suggesting intraventricular thrombus and severe systolic dysfunction. The patient received tirofiban intravenous therapy 48 hours after the procedure. Low molecular weight heparin was administrated following warfarin anticoagulation. The patient was discharged one day later on acetylsalicylic acid, beta-blockers, and angiotensin-converting enzyme inhibitor therapy.

## 3. Discussion

Mechanical heart valves are thrombogenic, and anticoagulation is essential to prevent thromboembolism and acute thrombotic obstruction [2]. Coronary artery embolism is not a rare cause of acute transmural myocardial infarction, representing an important entity in terms of etiology and clinical treatment [4], with incidence of 10 to 13% in autopsy series [5]. The consequences of myocardial infarction secondary to coronary artery embolism depend on both the size of the embolus and the luminal diameter of the vessel [5, 6].

Coronary embolism should always be suspected especially in the context of sudden chest pain in patients with valvular prosthesis, chronic atrial fibrillation, dilated cardiomyopathy, infective endocarditis, intracardiac shunts, cardiac myxoma, mural thrombi, and hypercoagulable states [1, 2, 7, 8]. It has also been observed that most emboli involve the left coronary system, which could be due to the preferential flow into the artery related to aortic valve morphology [9], as described in our case. However, there is no consensus on the best treatment for coronary embolism. 

When a patient presents early with myocardial infarction and ST segment elevation, thrombolysis or percutaneous transluminal coronary angioplasty are two options. Local thrombolysis can be considered but is cumbersome and often ineffective. However, some authors have considered the current medical approach with intravenous thrombolytic therapy [4, 10–12]. Although there is some evidence that a double regimen with thrombolytic agents is superior to a single regimen, published reports are few and the results are not definitive [7]. If the source of the embolus is infective vegetation, thrombolysis is contraindicated. There is a risk of distal embolization causing complete obstruction distally in a smaller branch [5]. 

Percutaneous transluminal coronary angioplasty and stenting may be used, as reported by Hernández et al. [13] in three interventional cases in which patients with coronary embolism and consequent acute myocardial infarction were treated successfully. In this context, Kiernan et al. [1] reported a 65-year-old male with mechanical aortic valve prosthesis and acute myocardial infarction due to coronary artery embolism. This individual decided to stop taking his warfarin considering previous periods involving bleeding possibly related to interaction between warfarin-metabolizing enzymes and taking a “herbal remedy” for pain causing an inappropriately high INR reading. Differently, in our case, the possible etiology of embolic manifestation was the concomitant presence of multiple thromboembolic risk factors, including smoking, using oral contraceptive pills and discontinued taking of warfarin.

Successful catheter aspiration embolectomy has been described by some authors [1, 7, 14, 15]. To prevent distal embolization, Belli et al. [14] and Beran et al. [15] described the use of commercial systems to aspirate thrombus from a native coronary artery and to improve the safety of epicardial flow. In our patient, catheter aspiration embolectomy for the intracoronary embolism was not effective. The complete distal obstruction of the left anterior descending coronary artery turned this procedure unfeasible. Still, the location of the coronary thrombus was a challenging place to reach and treat using percutaneous coronary intervention. In this case, anticoagulation with heparin and intravenous glycoprotein inhibitors was performed. This may not result in thrombus resolution but may prevent further increase in thrombus size [5]. It is also important to emphasize the need for aggressive anticoagulation, particularly in patients with mechanical prostheses, according to ACC/AHA guidelines [16].

The present report described a case of acute myocardial infarction due to coronary artery embolism in a smoker patient with mechanical aortic valve prosthesis who decided to abruptly discontinue her warfarin and started taking oral contraceptive pills three weeks earlier. It illustrates, once again, the risks of embolic events in high-risk patients, alerting for the hypothesis of embolic myocardial infarction in similar situations. In this context, the patient education on the optimal anticoagulation with warfarin and the medical advice about imminent thromboembolic risks are of extreme importance.
